# Maternal obese-type gut microbiota differentially impact cognition, anxiety and compulsive behavior in male and female offspring in mice

**DOI:** 10.1371/journal.pone.0175577

**Published:** 2017-04-25

**Authors:** Annadora J. Bruce-Keller, Sun-Ok Fernandez-Kim, R. Leigh Townsend, Claudia Kruger, Richard Carmouche, Susan Newman, J. Michael Salbaum, Hans-Rudolf Berthoud

**Affiliations:** Pennington Biomedical Research Center, Louisiana State University System, Baton Rouge, LA, United States of America; University of Missouri Columbia, UNITED STATES

## Abstract

Maternal obesity is known to predispose offspring to metabolic and neurodevelopmental abnormalities. While the mechanisms underlying these phenomena are unclear, high fat diets dramatically alter intestinal microbiota, and gut microbiota can impact physiological function. To determine if maternal diet-induced gut dysbiosis can disrupt offspring neurobehavioral function, we transplanted high fat diet- (HFD) or control low fat diet-associated (CD) gut microbiota to conventionally-housed female mice. Recipient mice were then bred and the behavioral phenotype of male and female offspring was tracked. While maternal behavior was unaffected, neonatal offspring from HFD dams vocalized less upon maternal separation than pups from CD dams. Furthermore, weaned male offspring from HFD dams had significant and selective disruptions in exploratory, cognitive, and stereotypical/compulsive behavior compared to male offspring from CD dams; while female offspring from HFD dams had increases in body weight and adiposity. 16S metagenomic analyses confirmed establishment of divergent microbiota in CD and HFD dams, with alterations in diversity and taxonomic distribution throughout pregnancy and lactation. Likewise, significant alterations in gut microbial diversity and distribution were noted in offspring from HFD dams compared to CD dams, and in males compared to females. Regression analyses of behavioral performance against differentially represented taxa suggest that decreased representation of specific members of the Firmicutes phylum predict behavioral decline in male offspring. Collectively, these data establish that high fat diet-induced maternal dysbiosis is sufficient to disrupt behavioral function in murine offspring in a sex-specific manner. Thus these data reinforce the essential link between maternal diet and neurologic programming in offspring and suggest that intestinal dysbiosis could link unhealthy modern diets to the increased prevalence of neurodevelopmental and childhood disorders.

## Introduction

A prevailing theory of neurodevelopmental pathogenesis is that environmental factors and genetic predisposition converge to disrupt neural circuits controlling social, emotional, and cognitive behavior [1],[2],[3],[4]. An environmental factor that could impair neurodevelopment is maternal obesity, as one third of women in the United States are obese [5]. The prenatal/perinatal environment plays a critical role in programming offspring’s metabolic and mental health; and an unhealthy maternal diet and/or maternal metabolic disorders (obesity, diabetes, hypertension, and pre-eclampsia) adversely impact offspring physiology and behavioral function [6],[7]. Although numerous experimental and epidemiological studies have confirmed an association between maternal obesity and adverse offspring outcomes, the underlying mechanisms remain unclear. In light of the coincident increases in obesity and neuropsychiatric disorders [8], it is crucial to understand the relationship between these two phenomena.

Diet-induced obesity could affect physiology through the gut microbiome, as modern diets high in fat and sugar are known to trigger robust and lasting alterations in intestinal microbiota [9]. The human gastrointestinal tract harbors a great number of bacteria from distinct species, and this dynamic population of microbes participates in physiologic functions including nutrition/digestion, growth, inflammation, immunity, and protection against foreign pathogens [10],[11],[12]. Accordingly, disruptions in the balance and diversity of intestinal microbiota could underlie variable host susceptibility to illness [13],[14], including neuropsychiatric impairment [15],[16]. Indeed, experimental and clinical studies strongly indicate that disruption to gut microbiota can impair brain function and mental health [11],[17],[18],[19],[20],[21],[22]. With regard to neurodevelopmental disorders, a number of conditions are associated with altered neonatal microbial colonization, which may impact intestinal barrier and immune maturation, the brain-gut axis, and ultimately contribute to development of neurologic disease [23],[24]. For example, autism spectrum disorders are strongly associated with gastrointestinal dysfunction [25], and collective studies reveal altered microbiota with shifts in several specific bacterial taxa in the stool of autistic children [26],[27],[28]. Furthermore, maternal nutrition during pregnancy may impact offspring gut microbiota and influence disease susceptibility [29]. While maternal obesity/high fat diet consumption has been linked to autism [30],[31],[32] and shown to modify offspring intestinal microbiota [33],[34], the direct link from unhealthy, high fat-shaped maternal microbiota to neurobehavioral impairment in offspring has only recently come under investigation [35] and remains incompletely understood. The aim of the present study was to test the hypothesis that high fat diet-associated maternal microbiota are sufficient to adversely affect neurobehavioral function in offspring. Adult female C57BL/6 mice maintained on chow diet were subjected to a microbiome depletion/transplantation paradigm using donor microbiota collected from either high fat diet- or control diet-fed mice [21]. Recipient female mice were then bred and the behavioral phenotype of male and female offspring tracked. Metagenomic sequencing of microbiota collected from dams and offspring at various times during pregnancy and postnatal development was conducted.

## Materials and methods

### Maternal microbiome transplantation, breeding, and behavioral testing

This study was carried out in strict accordance with PHS/NIH guidelines on the use of experimental animals, and the PBRC IACUC reviewed and approved all protocols. Microbiota donor material was taken from 4-month-old male C57Bl/6 mice (Jackson Laboratory) following 3 month exposure to either high fat diet (HFD; JAX # 380050, 60 kcal% fat (Research Diets, Inc. D12492i) or control diet (CD; JAX # 380056, 10 kcal% fat (Research Diets, Inc. D12450Bi). Cecal and colonic contents were harvested from donor mice, pooled, diluted 40-fold (weight: volume) in sterile water, and stored at -80°C as described previously [21].

**Fig 1:**
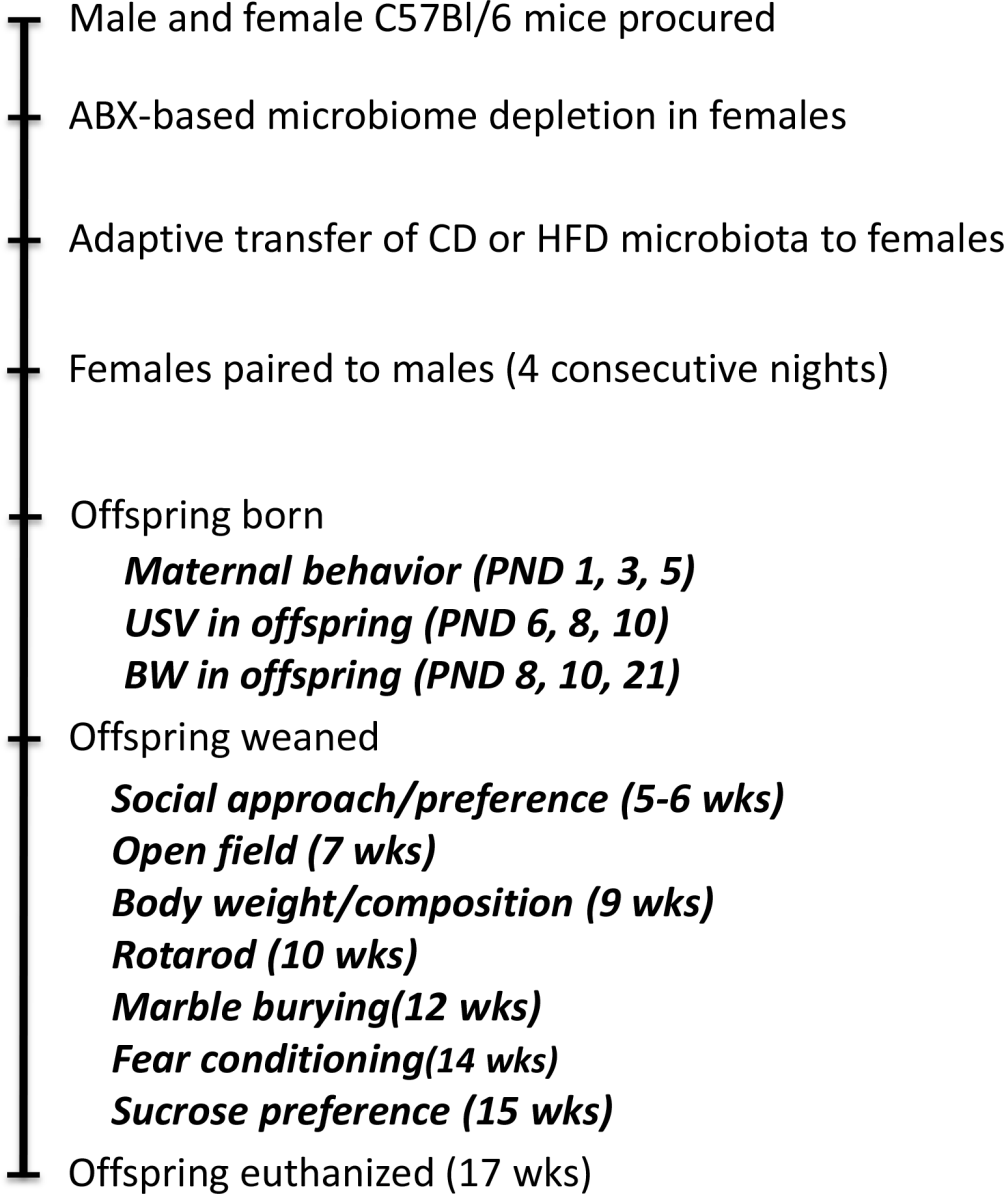
Schematic illustration of experimental design and schedule for behavioral testing. Adult (3 month old) male and proven breeder female C57Bl/6 mice purchased from Charles River Laboratories were single-housed under standard laboratory conditions. Female mice were subjected to the 14-day antibiotic-based microbiome depletion protocol, followed 48 hours later by transplantation with donor microbiota (daily gavage for 3 days, followed by twice-weekly boosts thereafter). Female mice were paired with males exactly 10 days after microbiome recolonization. Behavioral assessments were initiated sequentially in the order presented after litters were born, and the age of the mice when assessed is presented in parentheses (***PND*, *postnatal; wks*, *weeks)***.

The experimental timeline is schematically depicted in Fig 1. Adult (3 month old) male and proven breeder female C57Bl/6 mice (Charles River Laboratories) were single-housed under standard conditions with ad libitum access to water and “breeder chow”diet (Purina 5015; 19.7% of calorie provided from protein, 26.1% from fat, and 54.1% from carbohydrate). A cocktail of ampicillin, gentamicin, metronidazole, neomycin (all at 1.0 mg/day in sterile water), and vancomycin (0.5 mg/day) was given to female mice once daily for 14 consecutive days by oral gavage to deplete their native intestinal microbiome [36],[21]. The microbiome was re-established 72 hours after the last antibiotic administration via gavage administration of CD- or HFD-donor cecal contents (100 μl) daily for 3 days, followed by bi-weekly boosts (100 μl) throughout pregnancy and lactation. Females were paired with males exactly 10 days after the start of microbiome recolonization. Males were maintained under standard laboratory conditions with no microbiome manipulation, and were removed from the cages with females after 4 consecutive nights. Spontaneous maternal behavior in the home cage was assessed on postnatal days 1, 3, and 5 by real-time instantaneous sampling as described in S1 File.

### Offspring behavioral testing

All behavioral testing was conducted between 7 am and 1 pm, and was recorded/analyzed using Any-Maze software (Stoelting Co; see S1 File). Ultrasonic vocalizations from neonatal mice separated from their dams/littermates at postnatal days 6, 8 and 10 were recorded using an ultrasound microphone (Avisoft UltraSoundGate condenser microphone capsule CM16, Avisoft Bioacoustics, Berlin, Germany), and analyzed using Avisoft SASLab Pro (Version 5.0) [37]. Social approach/preference was assessed in weanling mice (3–4 week-old) using the standard three-chambered apparatus [38]. Overall anxiety and exploratory behavior was assessed using the Open Field assay [39]. Repetitive Motor Learning was assessed using a Five Station Rota-Rod Treadmill for Mouse (Med-Associates, St. Albans, VT) over 3 days (3 trials/day) with speed accelerating from 4 to 40 rpm. Stereotypical/compulsive behavior was assessed by quantifying marble burying during a 30 minute trial in a novel cage pre-loaded with 4 cm of clean bedding and 16 evenly-spaced marbles [40]. Sucrose preference/anhedonia was measured by offering mice a choice of 1% sucrose or water, with preference derived from the quotient of sucrose intake over total fluid intake. Both ad libitum intake and intake following a 4-hr fast was recorded. Memory was measured using a video-based fear conditioning system (Med-Associates) that pairs a unique context (scent and cage) and unconditioned stimulus (auditory tone) with a repeated foot shock (day 1), and then quantifies freezing behavior to the context (day 2) and to the tone (day 3) as a measure of memory [41].

### 16S metagenomic sequencing

Fecal samples were collected under aseptic conditions from dams before breeding, during pregnancy, and while nursing. Fecal samples were collected from offspring at weaning, while fecal and cecal samples were collected at euthanasia. DNA preparation, sequencing and bioinformatics were performed by the PBRC Genomics Core Facility. DNA was isolated using a commercial reagent system (MoBio Power Fecal Kit, MoBio Laboratories, Carlsbad, CA) augmented by enzymatic lysis using lysostaphin, mutanolysin, and lysozyme [42]. Sequencing libraries targeting V4 of the gene encoding the 16S ribosomal RNA were generated using a commercially available kit (NEXTflex™ 16S V4 Amplicon-Seq Library Prep Kit, BIOO Scientific, Austin, TX), relying of 16S gene-specific primer sequences V4F 5’-GTGCCAGCMGCCGCGGTAA-3’ and V4R 5’-GGACTACHVGGGTWTCTAAT-3’, and including Illumina adaptors and molecular barcodes as described by the manufacturer to produce 253bp amplicons. Samples were sequenced with custom primers (BIOO Scientific, Austin, TX) on an Illumina MiSeq instrument using version 3 sequencing chemistry (300bp paired end reads). Forward and reverse sequence reads were processed into double-stranded DNA contigs using quality control metrics implemented in the software package ‘mothur’ [43]. Sequence clustering (at better than 97% identity) to identify operational taxonomical units (OTU), removal of chimeric sequences, and generation of a read count table (i.e. tabulating the occurrence of each OTU in each sample) were performed with the software package ‘usearch ‘[44]. Taxonomical classification of each OTU sequence relied on the SILVA 16S rRNA sequence database version 123.1 [45], and statistical tests for differential representation were performed with tools incorporated in ‘mothur’, as well as using the software package DESeq2 [46]. Relative abundance of each OTU was examined on the phylum, class, order, family and genus levels.

### Statistical analyses

Behavioral and biochemical data were analyzed using Prism software (GraphPad Software, Inc.), and displayed as mean ± standard error. Body weight, maternal care behavior, ultrasonic vocalization, and fear conditioning data were analyzed with 2-way repeated measures ANOVA followed by planned Bonferroni post-hoc comparisons to determine differences between groups. All other behavior were analyzed by unpaired t-tests. Statistical significance for all analyses was accepted at p < 0.05, and *, **, and *** represent p < 0.05, p < 0.01, and p < 0.001, respectively. Alpha diversity (chao1 metrics) and beta diversity (weighted UniFrac metrics [47]) were assessed using tools implemented in ‘mothur’ on the basis of 80,000 sequences per sample. Beta-diversity between groups was also assessed by Per Mutational Multivariate Analysis Of Variance (PERMANOVA) to confirm group differences in overall microbiome composition. Differential representation of OTUs was assessed using DESeq2 on the basis of sequence count data, relying on Wald statistics with Benjamini-Hochberg correction and a false discovery rate cutoff set at 0.1. Inter-sample relationships relying on Principal Component Analysis on the basis of DESeq2 output, and data visualizations were both performed using JMP Genomics software (SAS, Cary, NC)

## Results

### Effects of intestinal microbiome transplantation on maternal body weight and behavior

**Fig 2:**
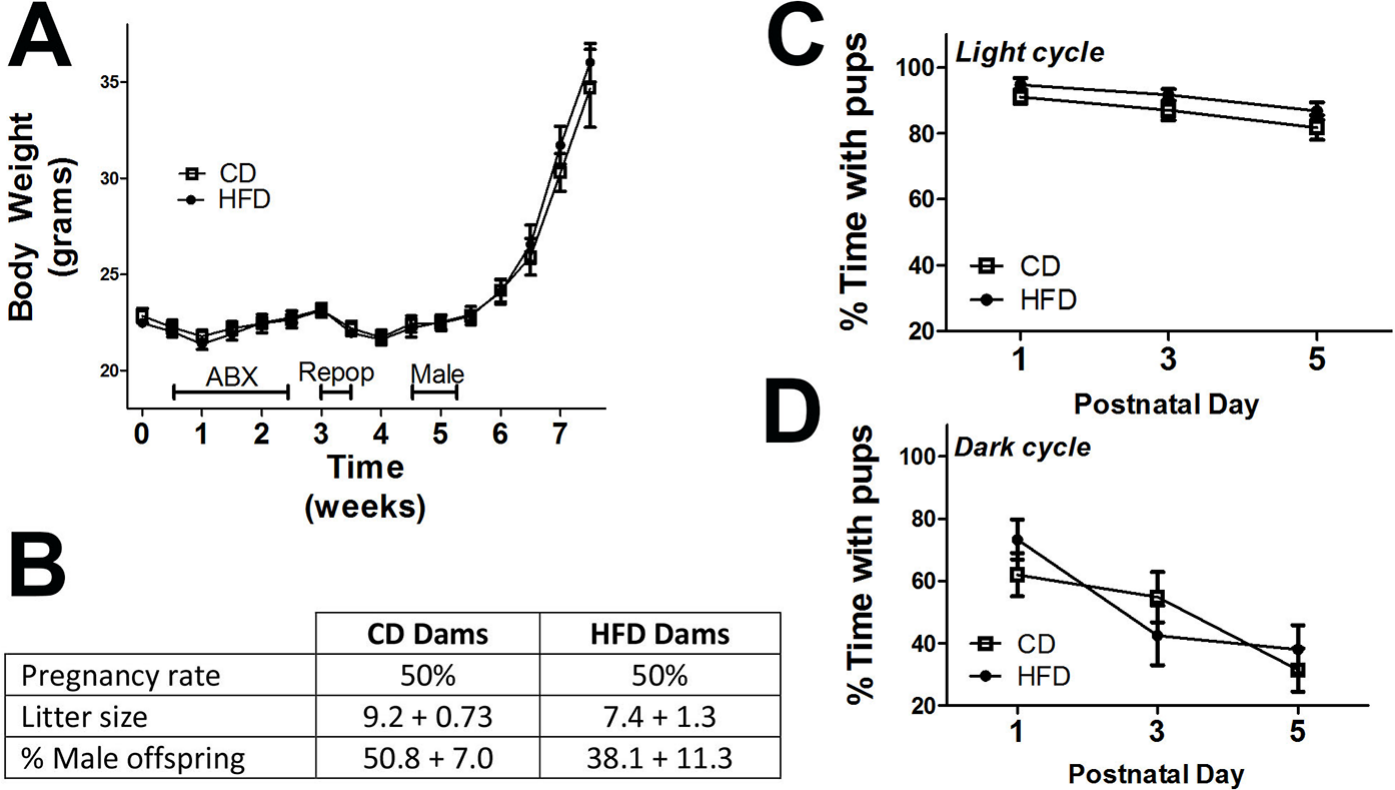
High fat diet associated microbiota do not affect body weight, pregnancy rates, or maternal care in breeding female mice. **(A)** Body weight of female mice during depletion (ABX), recolonization (Repop), breeding (Male), and pregnancy shows no difference between mice transplanted with microbiota from high fat diet fed donors (HFD) or control diet fed donors (CD). **(B)** Pregnancy rates following 4-night pairing with males, litter sizes, and sex balance of offspring in CD and HFD breeder dams. Data on litter size and % male offspring represent mean and SEM of 5 litters from each group of breeder mice, and were evaluated by unpaired t-test. Maternal behavior was assessed on postnatal days 1, 3, and 5, days, and was visually scored every minute for 1 hour during both light (9am) and dark cycles (9pm) as described in Methods. Individual behaviors were scored as “1” or “0”, and the following behaviors were counted: dam away from pups (grooming, eating or drinking, or sleeping); dam with pups (nursing pups in any posture, licking/grooming pups, nest-building or sleeping while in contact with pups) For presentation, maternal behavior data were summarized and presented as percent time with pups during either light **(C)** or dark **(D)** cycle.

All animals tolerated microbiome depletion/repopulation [36],[21] with only a temporary 5% loss of body weight (Fig 2A), and there were no differences in pregnancy rates, litter sizes, or sex balance of offspring between CD and HFD breeder dams (Fig 2B). More specifically, 10 proven breeder dams from each group (CD and HFD) were paired, resulting in 5 successful litters from each group, producing offspring in each group/sex ranging from 16 to 23 pups. Sample sizes (group/sex) were balanced to 15 mice after weaning, but offspring from only 4 CD and 4 HFD dams were followed into adulthood as not all litters contained both sexes. Spontaneous maternal behavior was assessed during both light and dark phases of postnatal days 1, 3, and 5, and for presentation, all maternal behavior data were summarized and presented as percent time with pups during either light or dark cycle. While nursing dams spent progressively less time physically in contact with pups as they grew, particularly during the dark cycle, no significant differences in time spent with pups between CD and HFD dams were noted (Fig 2C and 2D).

### Effects of maternal intestinal microbiome transplantation on offspring

#### Body weight and adiposity

Body weight during the postnatal period was measured beginning on postnatal day 8, and data show that female offspring from HFD dams were significantly heavier (*t*_(42)_ = 2.25, *p*<0.05) than females from CD dams at weaning (Table 1). As adults (9 weeks), female offspring from HFD dams remained significantly heavier (*t*_(42)_ = 2.15, *p*<0.05), and had significantly more body fat (*t*_(42)_ = 2.24, *p*<0.05) compared to adult females from CD dams (Table 1). No differences in body weight or composition in male offspring from CD or HFD dams were noted (Table 1).

**Table 1:** Body and fat weight in offspring from dams with CD- and HFD-shaped microbiota.

**Body Weight (gr)**	**From CD-Dams**	**From HFD-Dams**
Female PND 8	4.97 ± 0.10	5.43 ± 0.21
Female PND 10	5.98 ± 0.11	6.46 ± 0.25
Female PND 12	12.09 ± 0.22	12.86 ± 0.27*
Female Adult (9 wks)	22.72 ± 0.37	24.04 ± 0.51*
Male PND 8	5.02 ± 0.1	5.21 ± 0.1
Male PND 10	6.13 ± 0.1	6.23 ± 0.1
Male PND 12	12.78 ± 0.1	12.55 ± 0.1
Male Adult (9 wks)	28.68 ± 0.43	28.63 ± 0.68
**Total Body Fat (gr)**		
Female Adult (9 wks)	3.64 ± 0.25	4.48 ± 0.27*
Male Adult (9 wks)	4.67 ± 0.17	4.83 ± 0.43

Total body weight and body fat content in male and female offspring were measured on postnatal day (PND) 8, 10, and 12, and as adults (9 weeks old) as described in Methods. All offspring were maintained on chow diet. Data represent mean ± SEM, and were analyzed by 2-tailed, unpaired t-tests. Statistically significant increases in body weight and body fat in female offspring from HFD-dams as compared to female offspring from CD-dams mice are noted by *(p<0.05).

#### Ultrasonic communication

**Fig 3:**
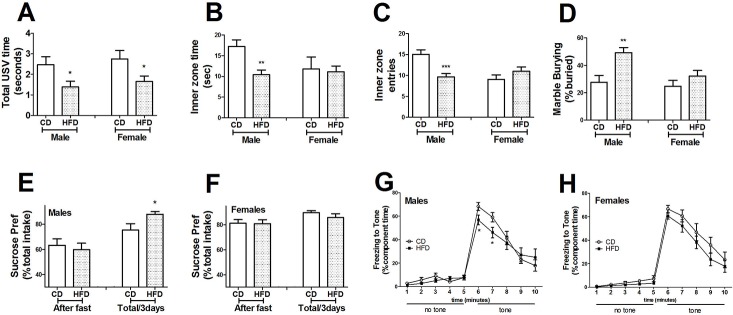
Maternal high fat diet associated microbiota causes sex-specific alterations in and behavioral outcomes in offspring mice. **(A)** Ultrasonic vocalizations (USV) were recorded over 2-minutes of separation from dam and littermates on postnatal day 8 as described in Methods. USV calls were reduced in male and female offspring of dams transplanted with high fat diet (HFD)-shaped microbiota offspring. **(B)** Analysis of behavior in the Open Field task revealed that male offspring from dams with HFD-shaped microbiota spent less time in the inner zone than male offspring from CD mice. **(C)** Entries into the inner zone of the Open Field were significantly decreased in male offspring from HFD mice as compared to male offspring from CD mice. **(D)** Stereotypical marble burying behavior was significantly increased in male offspring from HFD mice as compared to male offspring from CD mice. Preference for sucrose (1% in sterile water) over water was derived from the quotient of sucrose intake over total fluid intake, and both total ad libitum intake and intake following a 4-hr fast was recorded. Sucrose preference was significantly increased in male offspring **(E)** from HFD mice, but no changes in sucrose preference were noted in female **(F)** offspring. Following fear conditioning, conditioned freezing to the tone on day 3 was significantly reduced in male offspring **(G)** from HFD mice as compared to male offspring from CD mice, but no effect was noted in female **(H)** offspring. All data are presented as mean ± SEM of 15–20 mice per group, and * and ** indicate statistical significance (p < 0. 05, 0.01, respectively) based on unpaired t-tests or 2-way ANOVA.

Ultrasonic distress calls from pups isolated from their dam and littermates on postnatal days 6, 8 and 10 were recorded. Total call time (call number x duration) was significantly decreased in both male (*t*_(38)_ = 2.11, *p*<0.05) and female HFD (*t*_(43)_ = 2.21, *p*<0.05) offspring compared to CD offspring on postnatal day 8 (Fig 3A). There were no group- or sex- differences in total call time on postnatal days 6 or 10, and the ultrasonic frequency of calls emitted did not differ between groups, sex, or days (data not shown).

#### Exploratory and anxiety-like behavior

Exploratory and anxiety-based behavior was assessed using the Open Field assay. Male offspring from dams with HFD microbiota had significantly fewer entries into (t_(27)_ = 3.80, *p*<0.001), and spent significantly less time (*t*_(27)_ = 3.46, *p*<0.01) in the inner zone of the open field compared to male offspring from CD dams (Fig 3B and 3C). No differences in Open Field behavior were noted in female offspring (Fig 3B and 3C). No differences in mean speed or total distance traveled between CD and HFD groups of either sex were recorded (see S1 Fig), suggesting that the decreased exploratory behavior associated with males from HFD dams likely reflects increased anxiety and not decreased motor function.

#### Stereotypical behavior

Mice were then tested for stereotypical marble burying, a measure of agitated, compulsive, and repetitive behavior [48]. Male offspring from dams with HFD microbiota showed significant increases (t_(26)_ = 23.42, *p*<0.01) in marble burying, but no differences were noted in females (Fig 3D).

#### Sucrose preference

Preference/indifference for palatable foods in offspring was assessed by measuring sucrose preference under *ad lib* conditions and following a 4-hr fast. All animals preferred sucrose-water over plain water, but male offspring from HFD dams displayed a significant increase in sucrose preference under *ad lib* conditions compared to male offspring from CD dams (Fig 3D), while no differences between female groups were noted.

#### Auditory fear conditioning

Effects of maternal microbiota manipulations on offspring memory were assessed using the fear conditioning assay. Significant differences in freezing behavior were observed using the ‘‘tone test” as a measure of associative learning, and, post-hoc analyses revealed that freezing behavior in response to the tone was significantly decreased in male mice from dams with HFD microbiota as compared to male offspring from CD dams (Fig 3E), while no differences were noted in females (Fig 3F). While social approach/preference and repetitive motor learning were also measured, no group- or sex-differences were recorded (see S2 and S3 Figs).

### Phylogenetic profiles of intestinal microbiota from dams and offspring

#### Maternal metagenome

**Fig 4:**
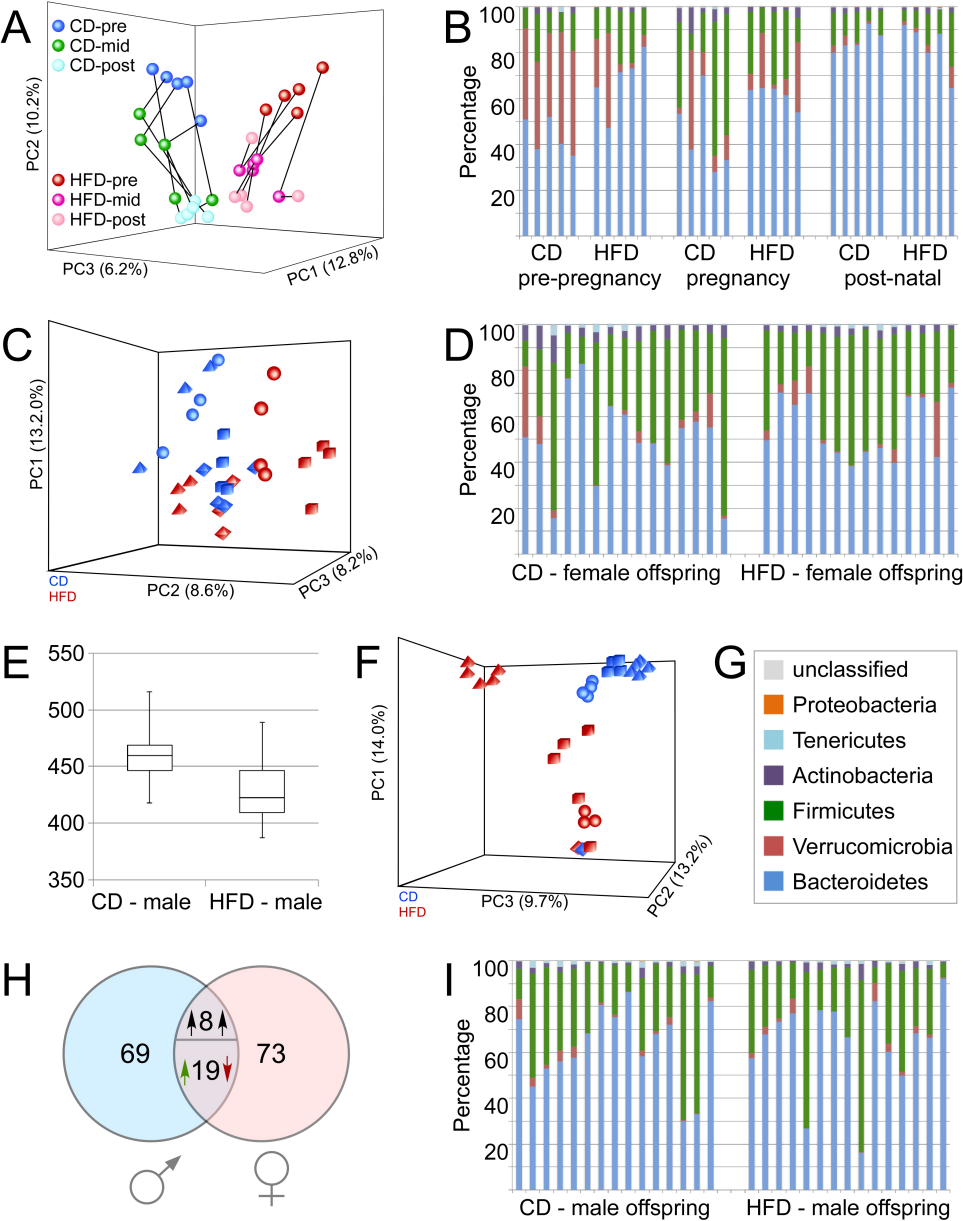
Effects of transplantation protocol on maternal and offspring microbiome. **A:** Normalized 16S metagenomics sequencing read count data obtained from fecal pellets of dams at three different time points were subjected to principal component analysis in order to reveal differences between groups. Samples from CD-reconstituted dams obtained pre-pregnancy (pre) at 2 weeks after antibiotic depletion and oral reconstitution (dark blue circles) form a distinct cluster compared to HFD-reconstituted dams (red circles). By mid-pregnancy (mid), changes are noted in community composition in both CD- (green) and HFD-reconstituted dams (magenta-pink). Samples obtained post-pregnancy (post) after weaning offspring show distinct clustering properties as well (CD: turquoise; HFD: pale pink). Distinction between the CD- and HFD-reconstituted dams is maintained along the entire timeline. Black lines show sequential development of community composition in individual dams. **(B)** Phylum-level community composition of CD- and HFD-reconstituted dams. HFD-reconstituted dams show a statistically significant increase of Bacteroidetes (blue) compared to CD-reconstituted dams (p = 0.007) at the expense of Verrucomicrobia (red; p = 0.009). No other statistically significant phylum-level differences were detected. **(C)** Gut microbiome sample relationships among female offspring from either CD- (blue) or HFD-reconstituted dams (red) were visualized using principal component analysis on normalized read count data generated by DESeq2 analysis for differential representation of individual taxonomical units. Different shapes (cubes, spheres, pyramids, and diamonds) identify female littermates that all came from the same dam in each group. **(D)** Phylum-level community composition in female offspring. **(E)** Alpha diversity analyses using chao1 metrics reveal significantly higher diversity (p = 0.009) in male offspring from CD-reconstituted dams compared to males from HFD-reconstituted dams. **(F)** Principal component analysis on normalized sequence count data shows significant differences in taxonomical representation in samples from male offspring of CD- (blue) and HFD-reconstituted (red) dams, suggesting that significant differences gut microbiome composition exist between the two groups. Different shapes (cubes, spheres, pyramids, and diamonds) identify male littermates that all came from the same dam. **(G)** Color legend for phylum-level community analyses in B, D, and I. **(H)** Comparison of sex-specific microbiome differences between offspring of CD- and HFD-reconstituted dams. In males, 96 OTUs are differentially represented (either enriched or depleted; FDR<0.1, more than 2-fold absolute fold change) in a HFD/CD comparison. In females, 100 OTUs fulfill this criterion. 27 OTUs are common in both sexes; 8 of those change concordantly, and 19 OTUs changing in a manner discordant between the two sexes. 69 OTUs are specific for the response in male offspring. **(I)** Phylum-level community composition in male offspring.

Analysis of dams and offspring fecal DNA via V4 16S rDNA sequencing identified a total of 708 operational taxonomical units (OTUs); 87% could be classified to family level, with 49.4% identifiable at genus level. In dams reconstituted with CD-microbiota, an average of 220 OTUs could be detected, while 214 OTUs were detected in HFD-reconstituted females. For all three time points (before mating, during pregnancy, during lactation), statistically significant differences in microbiota community composition were detected between CD- and HFD-reconstituted dams using weighted Unifrac phylogentic analysis tools (Table 2). However, no significant differences in alpha-diversity (Chao1 metric) were observed between CD- and HFD-reconstituted dams at any time (See S4 Fig). Visualization of beta-diversity via principal component analysis on normalized read count data showed group-based differences in beta-diversity of microbiota isolated at all 3 times points from breeding females (Fig 4A). Specifically, while both pregnancy and lactation introduced distinguishable shifts in community composition of both CD- and HFD-manipulated dams, the distinction between groups is maintained along the entire timeline despite changes between each time point group-differences persisted throughout pregnancy and lactation (Fig 4A). Statistically significant group-based differences in pre-pregnancy maternal microbiomes were confirmed by PERMANOVA analyses (F = 5.2, p = 0.009). The taxonomical distribution within groups at Phylum levels for the maternal fecal samples (Fig 4B) revealed that before pregnancy, HFD-reconstituted dams show a statistically significant elevated level of Bacteroidetes (blue) compared to CD-reconstituted dams (p = 0.007) at the expense of Verrucomicrobia (red; p = 0.009). No other phylum-level changes could be observed. Analyses at the level of individual OTUs demonstrated that 90 OTUs are differentially represented between CD- and HFD-reconstituted females (criteria: p_adj_<0.1, more than 2-fold absolute fold-change). The majority of these changes (87%) occur within the phylum Firmicutes (see S1 Table).

**Table 2:** Differences in microbiota community composition in dams with CD- and HFD-shaped microbiota and their offspring.

	Comparison	Score	P value
**Maternal Microbiota**	Pre-pregnancy: CD vs HFD	0.85984	<0.001
	Mid-pregnancy: CD vs HFD	0.623789	<0.001
	Lactation: CD vs HFD	0.543845	<0.001
		
**Offspring Microbiota**	Males: CD vs HFD	0.589994	<0.001
	Females: CD vs HFD	0.611843	<0.001
		
	CD: females vs males	0.698365	<0.001
	FDD: females vs males	0.655131	<0.001

Operational taxonomical units (OTU) were identified based on sequence clustering as described in Methods, and generation of a read count table was performed with the software package ‘usearch’. Statistical tests for differential representation were performed with tools incorporated in ‘mothur’, and statistically significant differences in microbiota community composition between groups were detected using weighted Unifrac phylogentic analysis tools.

#### Offspring metagenome

The microbiota of adult offspring was analyzed in cecal/colonic samples collected at euthanasia. Initial weighted Unifrac phylogentic analyses revealed statistically different microbiota composition between CD- and HFD-offspring of both sexes (Table 2). Significant sex-based differences were also noted in offspring from both CD- and HFD-manipulated dams (Table 2). There was no significant difference in alpha diversity in female offspring from CD compared with HFD dams (see S5 Fig). However, assessment of beta diversity in female offspring microbiota revealed significant differences between the two groups, as revealed by PERMANOVA analyses (F = 2.51, p = 0.003), and visualized on principal component analysis plots generated from normalized read count data (Fig 4C). Female littermates from individual HFD or CD dams could also be identified as groupings on principal component analysis plots, and PERMANOVA analyses likewise confirmed significant effects of kinship on beta diversity in female offspring (F = 3.17, p = 0.001).

Microbiota samples from female offspring of CD- and HFD-reconstituted females show significant group differences based on a weighted UniFrac test, and visualization of sample relationships via Principal Component Analysis based on normalized sequence count data shows that while there is a tendency towards formation of two clusters, there is considerable overlap between the two groups (Fig 4C). The taxonomical distribution at the phylum levels for the female offspring samples revealed no statistically significant differences (Fig 4D). Analysis of sequence count data via DESeq2 (relying on a statistical cutoff at p_adj_<0.1, and more than 2-fold absolute fold-change) demonstrated that 100 OTUs were differentially represented between female offspring of CD- and HFD-reconstituted dams, with 61 OTUs higher in females from HFD-dams, and 39 OTUs higher in offspring from CD-dams (see S2 Table). These analyses show that difference in the representation of individual OTUs drive group distinction in female offspring rather than phylogenetic differences.

Males born from CD-manipulated dams had significantly higher alpha diversity (Chao1, p<0.01) compared to male offspring from HFD dams (Fig 4E). Significant differences in beta diversity were also evident in males from CD dams relative to males from HFD dams as revealed by PERMANOVA analyses (F = 2.05; p = 0.034), and visualized on principal component analysis plots (Fig 4F). Similar to their female littermates, male pups born from individual HFD or CD dams could be identified in tight clusters on principal component analysis plots, and PERMANOVA likewise confirmed a significant and powerful effect of kinship in driving beta diversity in male offspring (F = 4.23; p = 0.001). Kinship effects persisted beyond weaning, as clusters of 5 or 6 are apparent (Fig 4C and 4F) while no more than 4 mice were ever group-housed post-weaning. Significant differences in phylum level taxonomical distribution were not observed for male offspring (Fig 4I), suggesting again that group distinctions are driven by individual OTUs. Accordingly, 96 individual OTUs were detected to be significantly different (p_adj_<0.1, more than 2-fold absolute fold-change) between male offspring from CD dams compared to males from HFD dams (see S3 Table). 27 of these OTUs were also found to be differentially represented in the two female offspring groups, yet only 8 OTUs displayed concordant (between sexes) differential (derived from HFD- vs. CD-reconstituted dams) representation; 19 OTUs show discordance between sexes. 69 OTUs exhibited differences only in male but not in female offspring (Fig 4H); overall, the male-specific pattern constitutes of 87 OTUs. The majority of male-specific changes (84%) occur within the phylum Firmicutes. Ten of these OTUs (OTU_022, Clostridium_sensu_stricto_1; OTU_023, Erysipelotrichaceae; OTU_045, Lachnospiraceae_NK4A136_group; OTU_054, Ruminococcaceae_UCG-014; OTU_081, Ruminococcaceae_UCG-013; OTU_090, Ruminococcaceae_UCG-014; OTU_175, Ruminococcaceae_UCG-014; OTU_183, Lachnospiraceae_NK4A136_group; OTU_266, Enterococcus; OTU_410, Lachnospiraceae_NK4A136_group) were also differentially represented in HFD- compared to CD-reconstituted dams before pregnancy, and we find a concordant direction of change (HFD vs. CD, dams and male offspring) for all but one OTU (OTU_022, Clostridium_sensu_stricto_1). This suggests that components of maternal microbiome constituents may contribute directly to the microbiome in male offspring.

A final analysis was undertaken to determine the relationship of specific intestinal taxa with behavioral function in male offspring. 23 individual OTUs that show log2-based group differences greater than 3 in male offspring (see S3 Table) were correlated against specific behavioral readouts. An additional 5 OTU’s for which the log2-fold change did not reach 3, but that were differentially expressed in both male offspring and in pre-pregnancy dams, were also selected (see S1 and S3 Tables). DESeq2 normalized read count values for these 28 OTUs were correlated against data from individual male mice subjected to ultrasonic vocalization (total calls), open field (time in inner zone), marble burying (% exposed), and fear conditioning (freezing at minute 6 or 7) assays to determine correlation coefficients (Pearson *r*) and p values (Table 3). Data show that 10 individual OTUs correlated significantly with at least 1 behavioral metric in male offspring, and that 9 of these OTUs arise from the Firmicutes phylum (Table 3). Importantly, nearly all of these taxa (9 of 10) correlate in a positive direction with behavioral data such that increasing scores reflect intact/appropriate function, meaning that decreased representation of these taxa is associated with behavioral dysfunction in male mice.

**Table 3:** OTUs whose representation predicts behavioral performance in male offspring.

		Correlation with Behavior in Male Offspring (Pearson *r* / p value)			
OTU	Log2FC HFD/CD	USV	OF	MB	FC
0054: Firmicutes/Clostridia/Clostridiales/Ruminococcaceae /Ruminococcaceae_UCG-014	• -2.12 • *-7*.*16 Dam*	*ns*	• 0.517 • p = 0.003	ns	• 0.571 • p = 0.002
0081: Firmicutes/Clostridia/Clostridiales/Ruminococcaceae /Ruminococcaceae_UCG-013	• -7.92 • *-9*.*99 Dam*	*ns*	*ns*	• 0.513 • p = 0.005	• 0.487 • p = 0.012
0109: Firmicutes/Erysipelotrichia/Erysipelotrichales /Erysipelotrichaceae	• -9.24	*ns*	• 0.442 • p = 0.014	• 0.529 • p = 0.004	• 0.446 • p = 0.022
0132: Firmicutes/Clostridia/Clostridiales/ Lachnospiraceae	• 17.49	*ns*	• -0.319 • p = 0.045	*ns*	*ns*
0152: Firmicutes/Clostridia/Clostridiales/Lachnospiraceae /Anaerostipes	• -3.95	*ns*	*ns*	• 0.409 • p = 0.034	• 0.450 • p = 0.021
0176: Tenericutes/Mollicutes/Mollicutes_RF9	• -8.46	*ns*	• 0.472 • p = 0.008	*ns*	• 0.438 • p = 0.025
0235: Firmicutes/Clostridia/Clostridiales/ Lachnospiraceae	• -3.86	*ns*	• 0.409 • p = 0.025	• 0.400 • p = 0.035	*ns*
0268: Firmicutes/Clostridia/Clostridiales/Ruminococcaceae /Ruminococcaceae_UCG-010	• -3.32	*ns*	• 0.552 • p = 0.002	*ns*	*ns*
0273: Firmicutes/Clostridia/Clostridiales /Lachnospiraceae	• -3.78	• 0.409 • p = 0.025	*ns*	*ns*	*ns*
0356: Firmicutes/Clostridia/Clostridiales/Lachnospiraceae /Lachnospiraceae_UCG-006	• -3.15	*ns*	*ns*	*ns*	• 0.428 • p = 0.029

Individual OTUs (taxonomic level as maximum taxonomical depth) that were differentially represented (increased or decreased) in HFD males as compared to CD males were correlated against behavioral performance. Log2-transformed fold-changes (Log2FC) in OTU representation are shown for male offspring, and for pre-pregnancy dams (*Dam*) if applicable. Data are correlation coefficients (Pearson *r*) and p values, and the specific behavioral metrics analyzed were ultrasonic vocalization (USV, total calling (duration*number)), open field (OF, time in inner zone), marble burying (MB, % exposed), and fear conditioning (FC, component freezing at minute 6 or 7) assays.

## Discussion

While adverse consequences of maternal obesity during pregnancy are well established, the mechanisms are unknown. Here we show that transplanting high fat diet-shaped gut microbiota into female mice before breeding is sufficient to disrupt neurobehavioral function in offspring. Specifically, neonatal offspring born from females with HFD-shaped microbiota emitted fewer ultrasonic calls upon separation, and also demonstrated sex-specific impairments in learning, anxiety, and compulsive-stereotypical behaviors later in life when compared to offspring from females with control-diet shaped microbiota. While ultrasonic vocalization was decreased in both male and female offspring, all other behavioral deficits were observed only in male offspring. Conversely, HFD-shaped maternal microbiota slightly increased body weight and adiposity in female, but not male offspring. Importantly, the transplant-recipient female dams were not obese or exposed to high-fat diet, and the HFD-shaped microbiome did not affect maternal contact or nesting behavior. These data suggest that altered gut microbiota are uniquely and intrinsically able to disrupt neurologic function in offspring. These findings are in agreement with the extensive body of literature documenting the adverse effects of maternal obesity and high fat diet consumption on body weight and neuronal function in offspring in both human and animal models [49],[50],[51],[52],[53]. For example, maternal high fat diet, obesity, and metabolic syndrome have all been shown to adversely impact the behavior and physiology of children [49, 51], [7],[31],[32]. In experimental laboratory settings, data link disruptions in social behavior, increases in anxiety-like and depressive-like behaviors, and impairments in cognition [54],[52, 55, 56] to maternal obesity. In light of increasing prevalence of both obesity[57] and neuropsychiatric disorders [58],[59] it is becoming increasingly important to resolve the relationship between these two concomitant public health crises.

### Role of maternal gut microbiota in altering behavior of offspring

Our data strongly suggest that diet-induced disruption to intestinal microbiota (gut dysbiosis) contributes to the adverse effects of maternal obesity on offspring. Intestinal microbiota are increasingly recognized as an important player in health and disease, and preclinical studies in rodents have clearly demonstrated that high fat diet-induced alterations to intestinal bacterial populations can cause metabolic and neurobehavioral impairment [21],[60]. In relation to maternal-fetal interactions, maternal obesity has been linked to altered offspring gut microbiota [34], which have in turn been linked to early manifestations of temperament in children [61]. Data also that the behavioral impairments in offspring of high-fat diet-induced obese mice can be transferred to germ-free mice [35], reaffirming a primary role of the gut microbiota. Furthermore, impairments in offspring can also be prevented by co-housing with offspring from healthy, regular diet-fed dams. Since mice are coprophagic, these results suggest that vertical transmission of unhealthy microbiota could mediate behavioral abnormalities in mice [35]. Indeed, our data indicate that maternal lineage is a powerfully strong diver of intestinal microbial composition in offspring, with kinship effects remaining strong even after weaning. While these data certainly imply that dietary and/or pharmacological disruption to healthy maternal gut microbiota could result in behavioral abnormalities associated with neurodevelopmental disorders, the mechanisms whereby maternal microbiota affect offspring remain unknown. There are multiple pathways that could facilitate bidirectional communication between gut microbiota and the brain, including direct interactions of microbes or microbial metabolites with intrinsic and extrinsic neurons, immune or inflammatory mediators produced by local immune tissues, or translocation of microbial products such as tryptophan metabolites or short-chain fatty acids into the circulation (reviewed in [62]). In terms of neurodevelopment, recent data suggest a key role for intestinal microbiota in the development of the CNS serotoninergic system. Specifically, data show that male mice born to germ-free dams have increased hippocampal 5-HT, decreased hippocampal BDNF, altered anxiety, and increased plasma levels of tryptophan [63]. While alterations in serotonin regulation in this study occurred only in males, other immunological and neuroendocrine consequences of germ-free development were evident in both sexes [63]. Collectively, these data suggest microbiota could modulate the development of CNS serotonergic neurotransmission by regulating peripheral precursors, but they also underscore the complexity of the likely bidirectional and sex-specific pathways whereby intestinal microbiota affect the developing brain.

### Sex differences in vulnerability to maternal manipulation

The demonstration of sex-specific responses to maternal microbiota is in keeping with an evolving understanding of the role of sex in developmental disorders. The concept of developmental programming posits that early life environment shapes offspring physiology throughout life, and sex-specific differences in offspring outcomes are becoming a standard metric of many developmental models [64]. Furthermore, the physiologic and behavioral outcomes of adverse *in utero* conditions appear more prominent in male than in female offspring [65],[66], suggesting that male fetal homeostasis may be more vulnerable to environmental influences. For example, environmental and olfactory stress during pregnancy disrupts emotionality in male but not female offspring [67], and maternal immune activation has likewise been shown to increase anxiety and depression specifically in male offspring [68],[69]. While the underlying molecular and cellular mechanism(s) whereby males are preferentially programmed remain unknown, the placenta is a widely recognized programming agent and data do suggest sex-specific alterations in placental homeostasis. For example, studies have noted that the placentas of female rodents respond more readily to prenatal insults [70],[71],[72], suggesting that female offspring are bolstered by enhanced placental resilience. Indeed, microarray data reveal sex-dependent differences in global transcriptomic profiles for human placenta, with females possessing more up-regulated autosomal genes, including immune-regulating genes [73], suggesting an enhanced response to perinatal infection. With regard to obesity, transcriptomic and epigenetic signatures also indicate that basal placental gene expression and responses to maternal high-fat diet are sexually dimorphic [74], [75].

### Specific microbiota participating in effects on offspring

It is unclear how specific microbiome constituents differentially influence offspring development. Several studies on obesity report reductions in Bacteroidetes and increases in Firmicutes [76],[77] that are reversed after weight loss [78],[79], indicating that the balance between these predominant phyla might impact host physiology. However, more recent data suggests that this binary distinction does not sufficiently reflect the complexity of high fat diet-induced alterations to the gut microbiome [80], [81], and that indeed, shifts *within* the Firmicute phylum might drive the overall distinction of high fat from control diet [21]. Data presented in this manuscript reinforces this view, as the vast majority (9 of 10) of the individual taxa that correlate with behavioral function come from the Firmicutes phylum, with decreased representation predicting impaired behavioral function. This suggestion of beneficial roles for representatives of the Firmicutes phylum is in keeping with the known ability of Firmicutes to produce butyrate as a byproduct of fermentation [82]. Butyrate is a major energy source for colonic epithelium [83], and more importantly, butyrate-producing bacteria play a vital role in maintaining the integrity of the intestinal epithelial barrier [84], [85]. Dysbiotic states with reduced butyrate production could facilitate translocation of intestinal antigens, which could disrupt brain function through multiple local or systemic pathways [86] [86]. Thus, the relative abundance of purportedly beneficial and harmful species in each group is likely to participate in neurobehavioral consequences rather than phylum-wide shifts in representation. However, another example is Verrucomicrobia, the phylum containing the presumed beneficial species *Akkermansia muciniphila*, which was significantly lower in dams following reconstitution with HFD-shaped microbiota, raising the possibility that loss of this species of bacteria may participate in the adverse effects of diet-induced dysbiosis, as suggested previously [21],[87]. The ongoing and collective identification of specific bacterial species/populations driving individual physiologic responses to diet could lead to personalized microbial therapies that optimize health in the context of modern diets/ lifestyles. Indeed, the use of gnotobiological methods on experimental animals has been indispensable in establishing the significance of specific microbiota taxa to mammalian physiology [88]. However, it is important to remember that germ-free mice are smaller than conventional mice, with decreased cardiac output and notably underdeveloped immune systems [89],[90]; and any of these confounds could affect pregnancy outcomes and fetal programming. Thus we feel that these data from transplantation models faithfully portray the essential link between maternal gut dysbiosis and neurologic outcomes in offspring. These data suggest that improved maintenance of healthy maternal microbiota, which is relatively inexpensive and uncomplicated, could potentially slow or reverse recent epidemiologic trends in neurodevelopmental childhood disorders.

## Supporting information

S1 File Supplemental Methods. (DOCX)

S1 Fig Open field speed and total distance traveled by offspring from dams with CD- and HFD-shaped microbiota. (DOCX)

S2 Fig 3-chamber assay for social preference in the same cohort of offspring from CD and HFD dams. (DOCX)

S3 Fig Motor coordination and learning in the same cohort of offspring from CD and HFD dams. (DOCX)

S4 Fig Fecal microbiota alpha-diversity from breeding female mice transplanted with CD- and HFD- shaped microbiota. (DOCX)

S5 Fig Fecal microbiota alpha-diversity in female offspring from female mice transplanted with CD- and HFD- shaped microbiota. (DOCX)

S1 Table Microbiome differences between breeding female mice with either CD- and HFD-shaped transplant. (DOCX)

S2 Table Microbiome differences between female offspring from dams with either CD- and HFD-shaped transplants. (DOCX)

S3 Table Microbiome differences between male offspring from dams with either CD- and HFD-shaped transplants. (DOCX)
