# Supplementary material for: Maternal obese-type gut microbiota differentially impact cognition, anxiety and compulsive behavior in male and female offspring in mice
Source: PLoS One. 2017 Apr 25;12(4):e0175577. doi: 10.1371/journal.pone.0175577 (PMC5404786; doi:10.1371/journal.pone.0175577)
Supplement: S1 File — (DOCX) [file pone.0175577.s001.docx]

**S1 File: Supplemental Methods**

***Details on behavioral tests*:** All behavioral testing was conducted between 7 am and 1 pm, and the sequence of individual tests was carried out in the specific order listed below to minimize carry-over effects across behavioral tests and prevent confounds caused by excessive handling or test-associated stressors. All behavioral procedures were video-recorded and analyzed using ANY MAZE software (Columbus Instruments, Ohio, US), providing unbiased quantification of animal behavior, including body location, orientation, distance, speed, and mobility/immobility.

*Maternal care behavior:*  Maternal care was visually scored every minute for 1 hour during both light (9am) and dark cycles (9pm), for a total of 360 observations per dam (60 observations/period×2 periods/day×3 observation days). Individual behaviors were scored as “1” or “0”, and the following behaviors were counted: dam away from pups (grooming, eating or drinking, or sleeping); dam with pups (nursing pups in any posture, licking/grooming pups, nest-building or sleeping while in contact with pups).

*Ultrasonic vocalizations:* Neonatal mice were permanently identified by amputation of a distal phalanx from either the left or right foot on postnatal day 5, and 5-minute recordings from individual were taken from mice that have been removed from their home cages postnatal day 6, 8 and 10 and temporarily (exactly 3 minutes) placed in dark, sound-isolating chambers. Ultrasonic vocalizations (total number of calls, average call duration, peak frequencies, and peak amplitude) were recorded for 2 minutes after a 1-minute acclimation period using an Ultrasound Microphone (Avisoft UltraSoundGate condenser microphone capsule CM16, Avisoft Bioacoustics, Berlin, Germany), and analyzed using Avisoft SASLab Pro (Version 5.0). Frequency sonograms were prepared from recordings (frequency range = 40 kHz to 120 kHz, FFT size = 256, overlap = 50%), and trained, blinded investigators manually scored total number of calls, average call duration, peak sound frequencies, and peak amplitude at the peak frequency.

*Social approach/preference:* Weanling mice (5-6 week-old) were tested for social preference using the standard three-chambered apparatus containing inverted wire cups (Galaxy Cups, Kitchen Plus, <http://www.kitchen-plus.com>). Briefly, test mice were allowed to habituate to the center chamber for 5 min, followed by 5 min of free exploration of all chambers to confirm the absence of a side preference. Following acclimation, an age- and sex-matched partner mouse was placed in one cup and an object of approximately the same size in other cup, and the test mouse was returned to the center chamber with both doors open for a 10 min session (session 1), followed by an additional 10 minute session with the object replaced by another unknown mouse (session 2). Video tracking was used to quantify the amount of time spent in each chamber, the amount of time physically interacting or sniffing within a 2 cm radius of the target, and the number of entries into each chamber.

*Open Field test:* Overall motor and exploratory behavior was quantified using standard open field procedures. Each mouse was placed in the center of the open box (50x50 cm) and allowed to freely explore for 10 min. Total time spent in each the outer (10 cm from edge), inner (inner 10 cm), and middle (between inner and outer) zones was quantified, as was total overall distance traveled and mean velocity.

*Rotarod*: testing of psychomotor function and coordination was conducted using a Five Station Rota-Rod Treadmill for Mouse (Med-Associates, St. Albans, VT). Each mouse was given 3 trials/day over 3 days, with the starting speed of the rotarod 4 rpm accelerating to 40 rpm over a period of five min. The amount of time the animal was able to remain on the accelerating rotating cylinder (latency to fall) was recorded, and the maximum trial length was 5 min with a 30 min rest period between each trial.

*Marble burying test:* Stereotypical burying behavior was quantified in plastic test cages with 4-cm of bedding and 16 sterilized glass marbles are spaced evenly apart. Mice were removed after 30 minutes, and the total surface area of marbles visible from above was quantified via image analysis.

*Sucrose preference/anhedonia.* Mice were temporarily switched to single occupant cages with two identical volumetric drinking tubes containing either sterile water or 1% sucrose in sterile water.  Bottles were weighed to measure fluid intake during every 24 hrs, and the position (right v. left) was alternated each period to eliminate potential bias from place preference.  After 24 hrs of acclimation, baseline preference was measured every 24 hours for 2 consecutive days.  Mice were then briefly deprived of water and food for 4 hrs (from 13:00 PM to 17:00 PM), followed by a 1-hour preference test with water and 1% sucrose. The bottles containing water and sucrose were weighed before and after, and sucrose preference (%) was calculated using the following formula: [Sucrose intake/(Water intake + Sucrose intake)] × 100.

*Fear conditioning memory task:* Each mouse was individually evaluated for fear conditioning using an automated, video-based fear conditioning system (Med-Associates, St. Albans, VT). The fear conditioning assay is a type of associative learning task in which subjects are presented with a neutral stimulus (tone) that is paired with an aversive unconditioned stimulus (foot shock), subjects display fear behavior (freezing) in response to the tone. The apparatus consists of a “startle chamber” used on days 1 and 2, which is an 8×15×15-cm acrylic and wire mesh cage located within a custom designed 90×70×70 ventilated sound-attenuating chamber. The floor of each chamber is made of 2.0-mm diameter stainless steel bars spaced 6 mm apart, through which shock is administered, and the unique context is reinforced with an anise-based scent applied to each cage before testing. Animal movement within the apparatus results in displacement of an accelerometer (model U321AO2; PCB Piezotronics, Depew, NY, USA) with the resulting voltage being proportional to the velocity of displacement. For day 3, an entirely separate chamber located in a different room is used to remove all contextual cues. These “trace” chambers contain a flat floor instead of the grid, a ”teepee”-shaped insert to modify the chamber dimensions, and an acetic acid odor used to reinforce the novel environment.

Acquisition of fear conditioning on day 1 consisted of 5 minutes of acclimation to the startle chamber, followed by five consecutive 30 second auditory stimuli (85 db, 4 KHz) that co-terminated with a mild footshock (0.5 mA × 1 sec), with 30 second recovery periods between tones. On day 2, the mice were returned to the same chambers, but no stimuli were applied to evaluate freezing responses to context. On day 3, the mice were placed in the novel “trace” chambers, and after habituation of the mice within the chambers for 5 minutes, a continuous tone (85 db, 4 KHz) was applied for 5 minutes. The percent of freezing was recorded as a measure of trace memory of the conditioned response to the tone.
