# Supplementary material for: Maternal obese-type gut microbiota differentially impact cognition, anxiety and compulsive behavior in male and female offspring in mice
Source: PLoS One. 2017 Apr 25;12(4):e0175577. doi: 10.1371/journal.pone.0175577 (PMC5404786; doi:10.1371/journal.pone.0175577)
Supplement: S1 Fig — (DOCX) [file pone.0175577.s002.docx]

**S1 Fig**


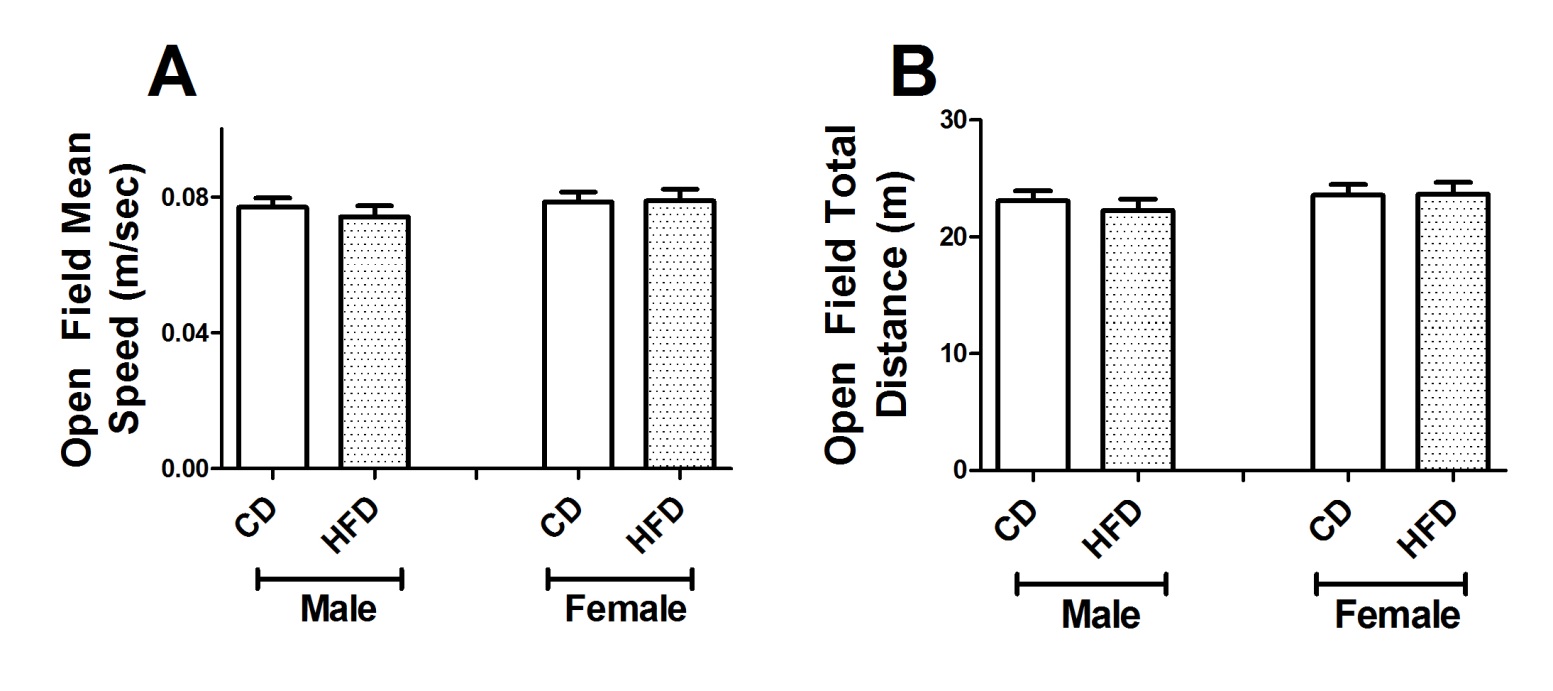


**Supplemental Figure 1. Open field speed and total distance traveled by offspring from dams with CD- and HFD-shaped microbiota.** Male and female offspring of dams with HFD- or CD microbiota were assessed in the Open Field assay. Data are mean±SEM of mean speed (A) and total distance traveled during the 5 minute trial (n

=

15 per group).
