# Supplementary material for: Maternal obese-type gut microbiota differentially impact cognition, anxiety and compulsive behavior in male and female offspring in mice
Source: PLoS One. 2017 Apr 25;12(4):e0175577. doi: 10.1371/journal.pone.0175577 (PMC5404786; doi:10.1371/journal.pone.0175577)
Supplement: S2 Fig — (DOCX) [file pone.0175577.s003.docx]

**S2Fig**

**
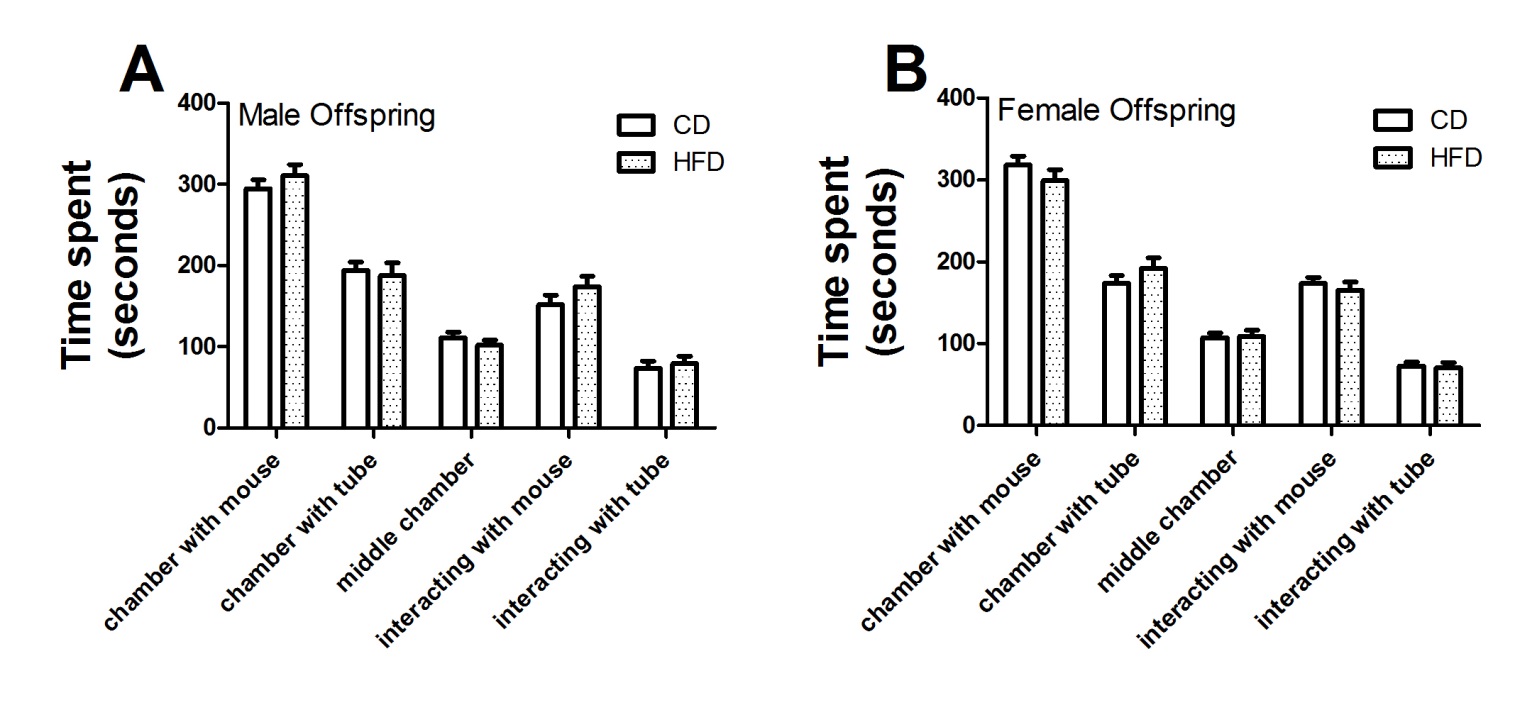
**

**Supplemental Figure 2. 3-chamber assay for social preference in the same cohort of offspring from CD and HFD dams.** Male and female offspring of dams with HFD- or CD microbiota were assessed using the 3-chamber assay for social approach and preference. Data are mean±SEM (n=15) of % time spent in the chamber with the mouse (“chamber with mouse”), the chamber with an inanimate object (“chamber with tube”), or the middle chamber. Additionally, %time spent physically interacting with the mouse (“interactive with mouse”) or the inanimate object (“interactive with tube”) was assessed.
