# Supplementary material for: Maternal obese-type gut microbiota differentially impact cognition, anxiety and compulsive behavior in male and female offspring in mice
Source: PLoS One. 2017 Apr 25;12(4):e0175577. doi: 10.1371/journal.pone.0175577 (PMC5404786; doi:10.1371/journal.pone.0175577)
Supplement: S3 Fig — (DOCX) [file pone.0175577.s004.docx]

**S3. Fig**

**
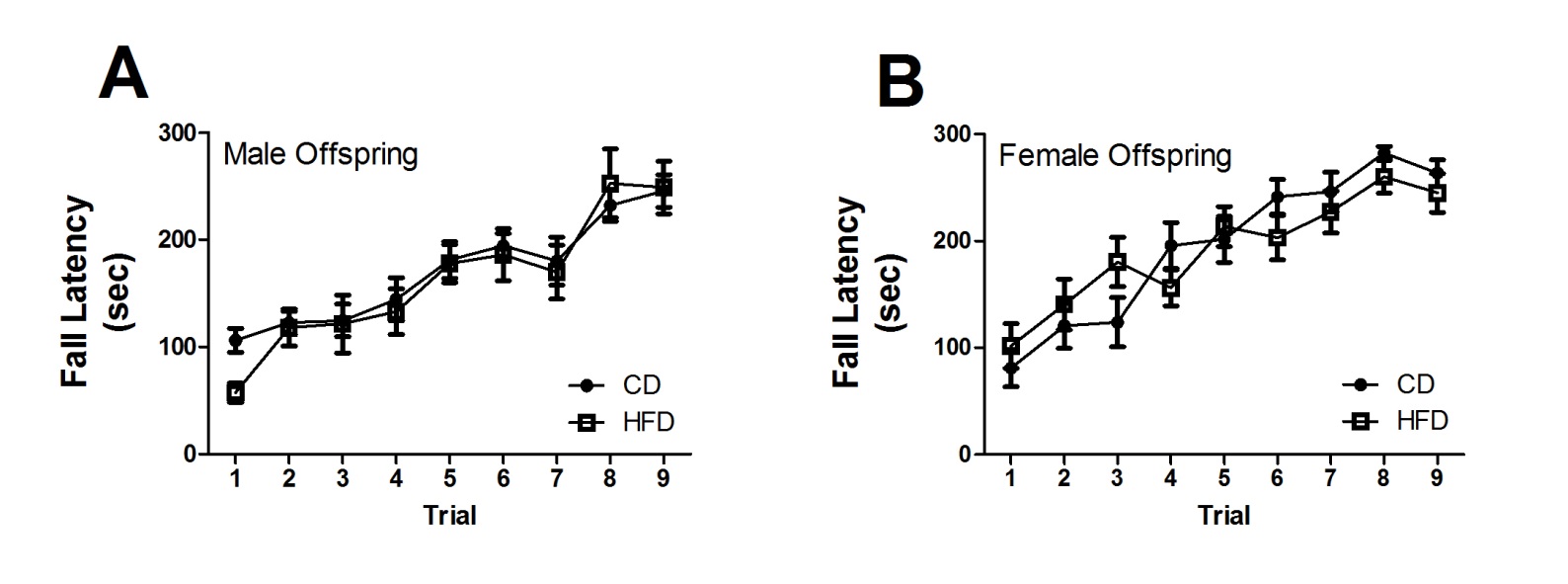
**

**Supplemental Figure 3. Motor coordination and learning in the same cohort of offspring from CD and HFD dams**. The latency to fall from an accelerating rotarod was measured by 3 trials per day over 3 consecutive days in the rotarod test. There was no significant difference between male (A) or female (B) offspring groups in in fall latency.
