# Supplementary material for: Maternal obese-type gut microbiota differentially impact cognition, anxiety and compulsive behavior in male and female offspring in mice
Source: PLoS One. 2017 Apr 25;12(4):e0175577. doi: 10.1371/journal.pone.0175577 (PMC5404786; doi:10.1371/journal.pone.0175577)
Supplement: S5 Fig — (DOCX) [file pone.0175577.s006.docx]

**S5 Fig**

***
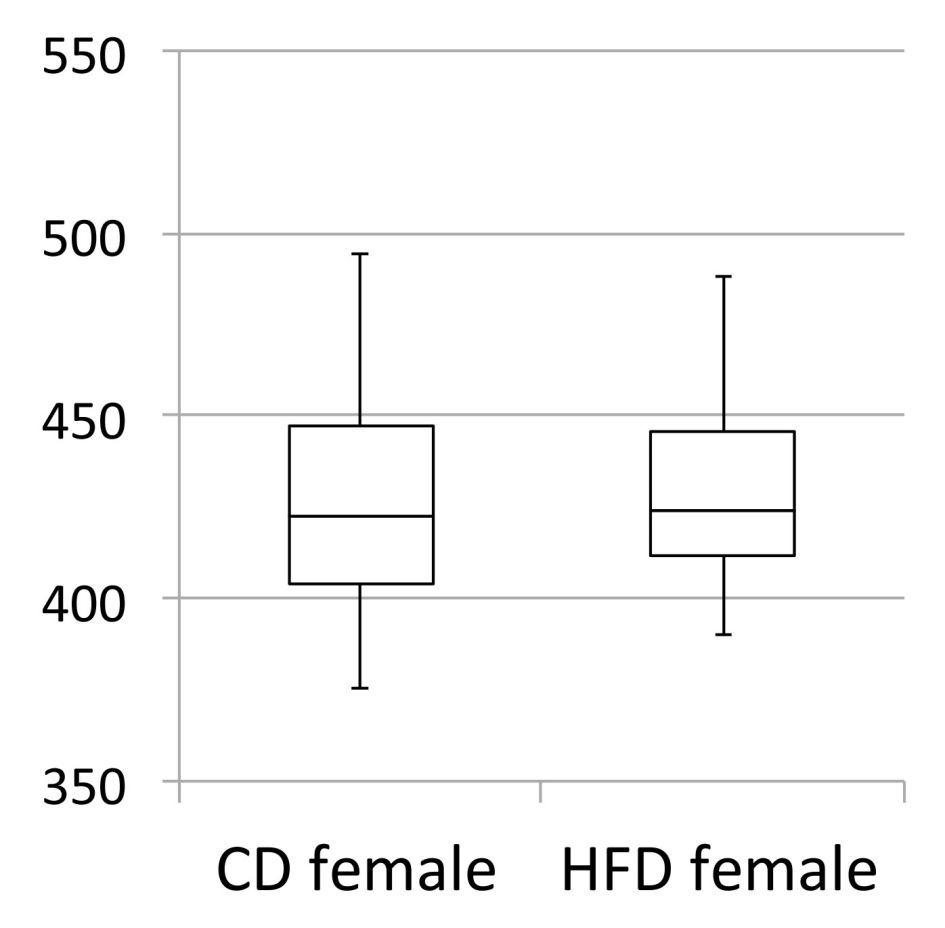
***

**Supplemental Figure 5. Fecal microbiota alpha-diversity in female offspring from female mice transplanted with CD- and HFD- shaped microbiota.** Chao1 and Observed Species Box Plots depict no statistically significant differences in alpha-diversity between female offspring of CD- and HFD-reconstituted dams.
