# Supplementary material for: Maternal obese-type gut microbiota differentially impact cognition, anxiety and compulsive behavior in male and female offspring in mice
Source: PLoS One. 2017 Apr 25;12(4):e0175577. doi: 10.1371/journal.pone.0175577 (PMC5404786; doi:10.1371/journal.pone.0175577)
Supplement: S1 Table — (DOCX) [file pone.0175577.s007.docx]

**S1 Table**

**
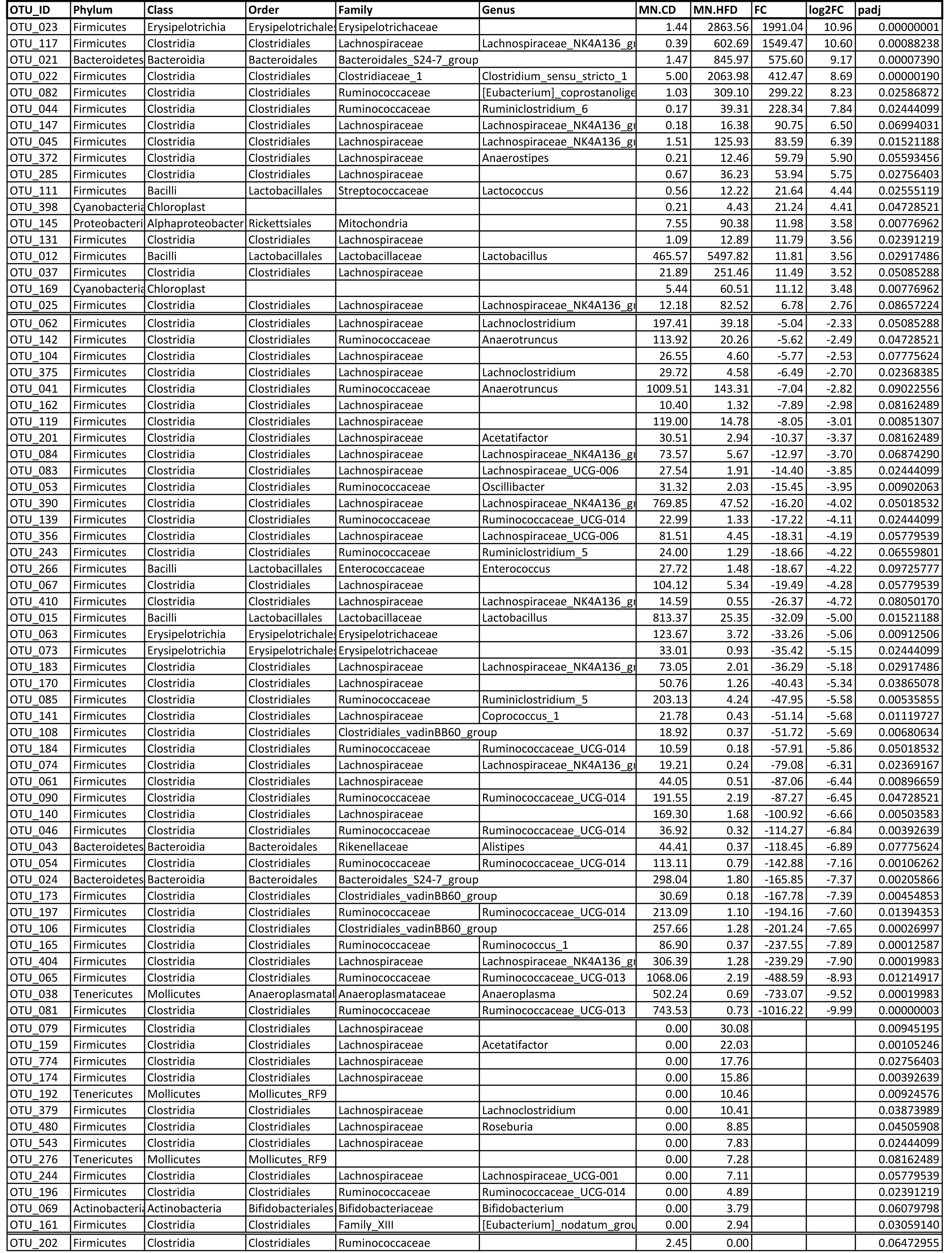
**

**
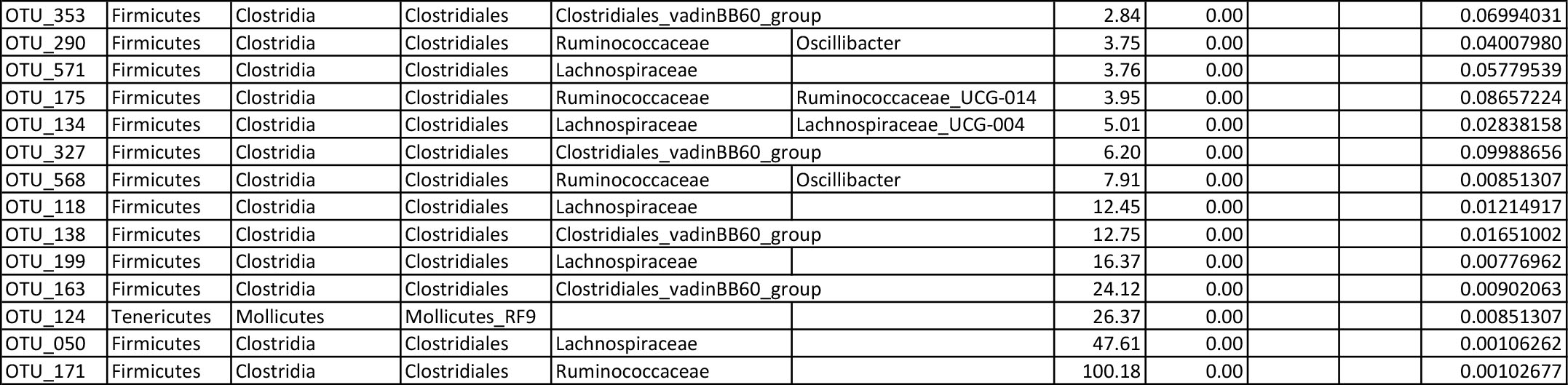
**

**Supplemental Table 1: Microbiome differences between breeding female mice with either CD- and HFD-shaped transplant**. Individual microbiome constituents with statistically significant (padj<0.1) enrichment/depletion-based group-differences in dams after microbiome reconstitution immediately before breeding were determined in DESeq2. Significant fold-changes greater than 2 (|FC|>2) for individual OTUs (taxonomic level as maximum taxonomical depth) were log2-transformed and plotted relative to the CD group.
