# Supplementary material for: Maternal obese-type gut microbiota differentially impact cognition, anxiety and compulsive behavior in male and female offspring in mice
Source: PLoS One. 2017 Apr 25;12(4):e0175577. doi: 10.1371/journal.pone.0175577 (PMC5404786; doi:10.1371/journal.pone.0175577)
Supplement: S2 Table — (DOCX) [file pone.0175577.s008.docx]

**S2 Table**


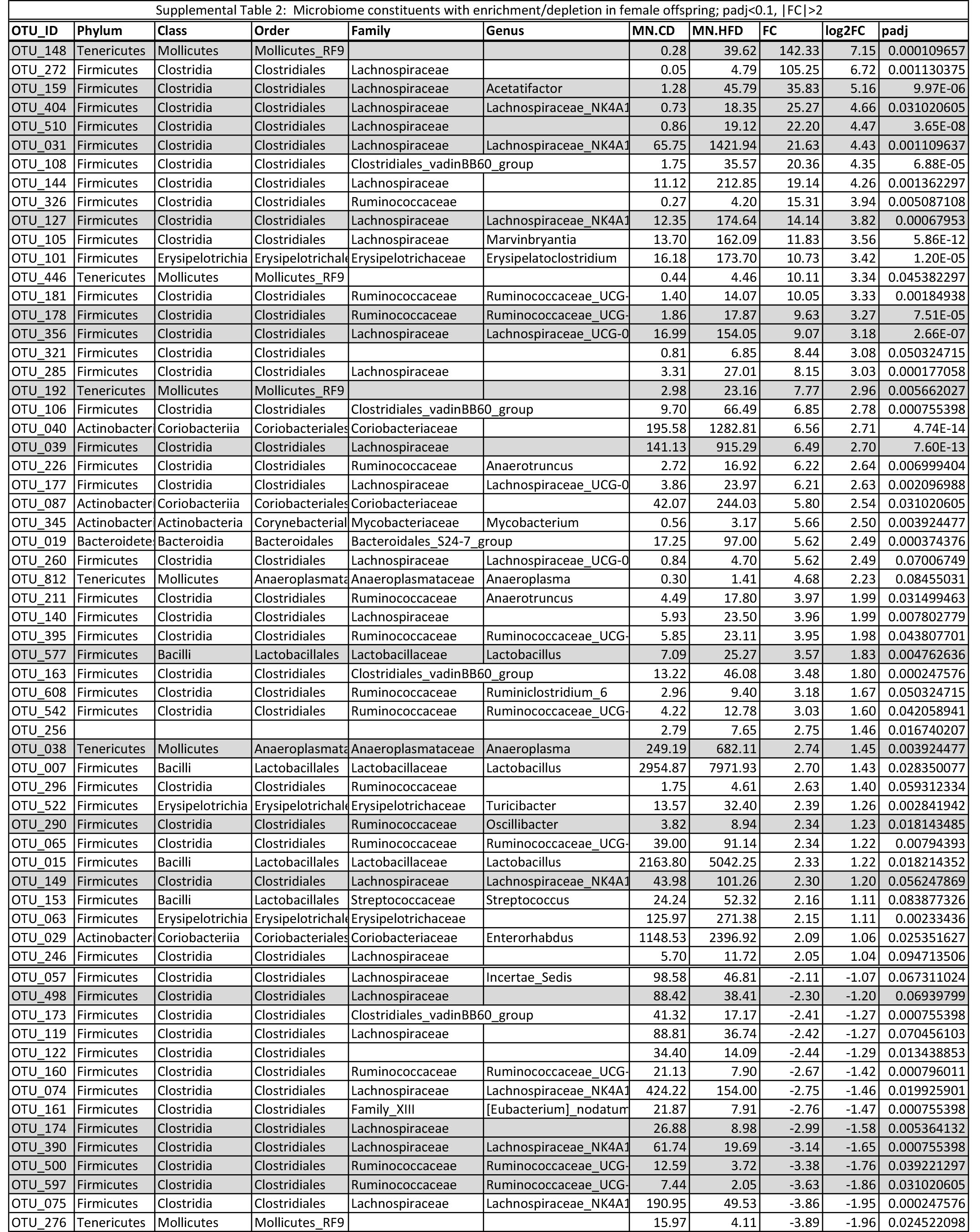


**
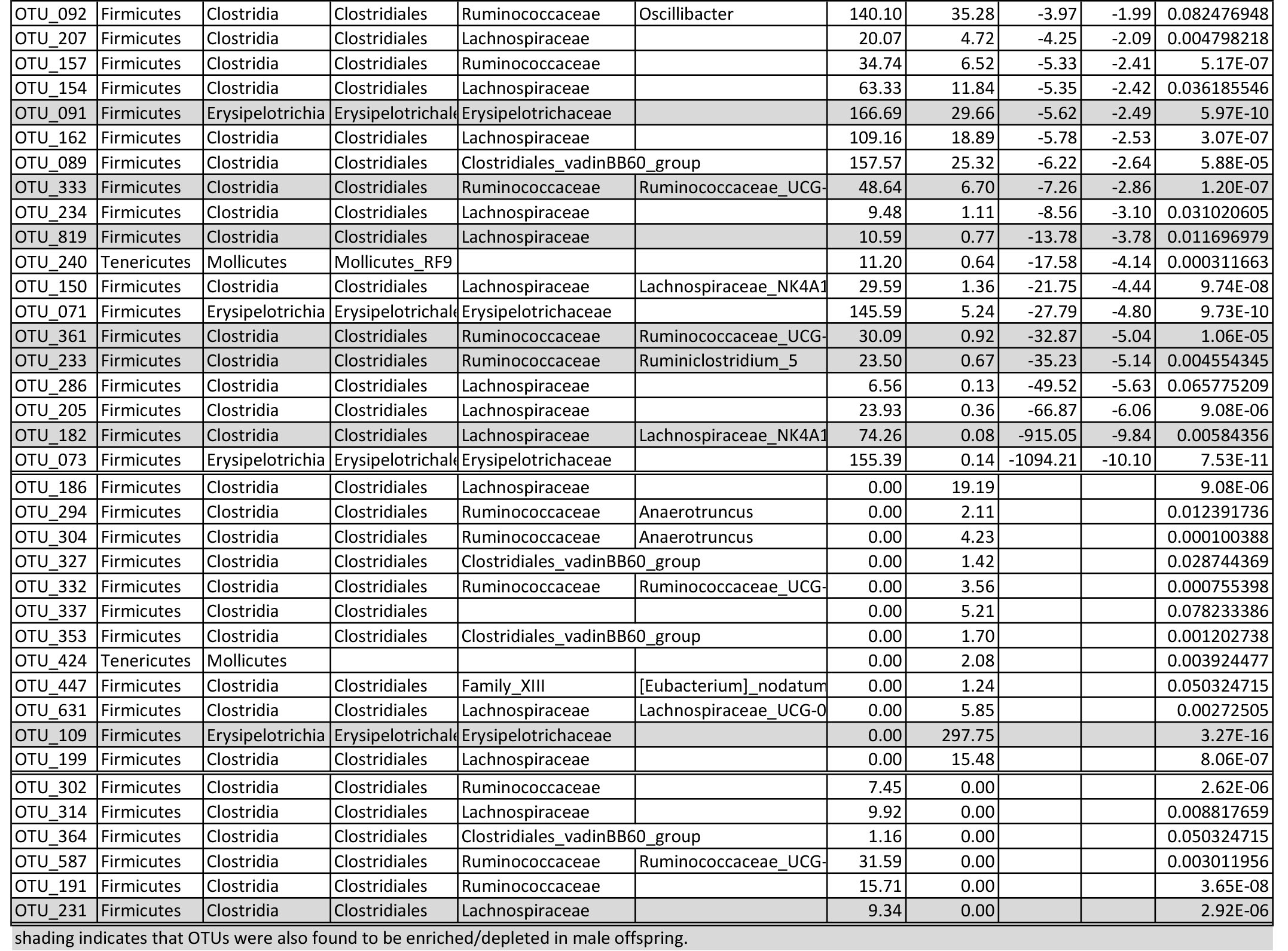
**

**Supplemental Table 2: Microbiome differences between female offspring from dams with either CD- and HFD-shaped transplants**. Individual microbiome constituents with statistically significant (padj<0.1) enrichment/depletion-based group-differences in female offspring were determined in DESeq2. Significant fold-changes greater than 2 (|FC|>2) for individual OTUs (taxonomic level as maximum taxonomical depth) were log2-transformed and plotted relative to the CD group. Shading indicates that OTUs were also found to be enriched/depleted in male offspring.
